# Transcending Erotomania: A Case of Long-Term Multi-Comorbid Management in an Adolescent Medium Secure Unit

**DOI:** 10.1192/bjo.2024.688

**Published:** 2024-08-01

**Authors:** Manzar Shahid, Parag Shah, Muhammad Saqib Siddique

**Affiliations:** Cumbria, Northumberland, Tyne and Wear NHS Foundation Trust, Newcastle upon Tyne, United Kingdom

## Abstract

**Aims:**

20 year old patient open to mental health services since the age of 8. Through the years, they have had work-up and diagnoses of ADHD, ASD, Schizoaffective Disorder (with prominent erotomanic delusions) and sexual identity concerns.

They spent a number of years in psychiatric in-patient units following an index offence. Initially in an adolescent LSU and subsequently in an adolescent MSU.

After 5 years of their stay at the MSU, their transition to an adult rehabilitation ward was planned and completed.

**Methods:**

20 year old, oldest of 5 siblings, born with no complications during or after pregnancy and at full-term. First referred to mental health services aged 6 regarding difficulties at school leading to a diagnosis of ADHD.

At age 13, re-engaged with mental health services following concerns around self-harm, disappearing from home, alcohol use. Also with difficulties around gender identity and sexual orientation. Shortly after, elements of ASD were identified, including social and communication difficulties and special interests which included single females.

Around age 16, patient developed erotomanic delusions. First towards a female friend in dance class – patient wanted to run away with them and have their babies, and carried a knife to hurt anyone who tried to get in their way, eventually leading to the index offence. In addition, there were similar erotomanic delusions with regards to at least 2 famous female music personalities.

With a significant mood component accompanying the psychosis, she was diagnosed and managed as having Schizoaffective Disorder.

**Results:**

The patient presented with a complex, multimodal presentation which took time and a comprehensive holistic approach. They were trialled on 3 different antipsychotics and eventually clozapine which needed stopping due to side effects. Best response was eventually observed with a return to olanzapine.

Patient also had 19 treatments of ECT (13 being high dose) with marked transient improvement.

Psychology, OT and the MDT largely focussed on building therapeutic relationships with the patient which gradually helped the patient develop insight around their erotomanic delusions and the impact on their life.

**Conclusion:**

Despite the complexities of this case, it highlighted that a robust, consistent, holistic approach can change lives even though this may take some time. The patient was utilizing leaves off the ward, taking part in the education sessions and activities on the ward and has recently been transferred to an adult rehabilitation ward after years in an adolescent specialist in-patient service.

